# When clinicians and patients disagree on vaccination: what primary care clinicians can learn from COVID-19-vaccine-hesitant patients about communication, trust, and relationships in healthcare

**DOI:** 10.1186/s12875-024-02665-1

**Published:** 2024-12-05

**Authors:** Natalie Purcell, Hajra Usman, Nicole Woodruff, Haley Mehlman, Leah Tobey-Moore, Beth Ann Petrakis, Karen Anderson Oliver, Adam Kaplan, Jeffrey M. Pyne, Jennifer K. Manuel, Beth M. DeRonne, Dan Bertenthal, Karen H. Seal

**Affiliations:** 1 San Francisco Veterans Affairs Health Care System, San Francisco, CA USA; 2 Department of Social and Behavioral Sciences, University of California, San Francisco, CA USA; 3 Northern California Institute for Research and Education, San Francisco, CA USA; 4 Keck School of Medicine, University of Southern California, Los Angeles, CA USA; 5 Department of Psychology, University of Michigan, Ann Arbor, MI USA; 6 Center for Mental Healthcare and Outcomes Research, Central Arkansas Veterans Healthcare System, Little Rock, AR USA; 7 Department of Psychiatry, University of Arkansas for Medical Sciences, Little Rock, AR USA; 8 Department of Geriatrics, University of Arkansas for Medical Sciences, Little Rock, AR USA; 9 Center for Health Optimization and Implementation Research, Veterans Affairs Bedford Health Care System, Bedford, MA USA; 10 Center for Care Delivery and Outcomes Research, Minneapolis Veterans Affairs Health Care System, Minneapolis, MN USA; 11 Department of Psychiatry and Behavioral Sciences, University of California, San Francisco, CA USA; 12 Department of Medicine, University of California, San Francisco, CA USA

**Keywords:** COVID-19 vaccination, Vaccine hesitancy, Healthcare communication, Healthcare relationships, Motivational interviewing, Trust

## Abstract

**Background:**

In the United States, discourse on COVID-19 vaccination has become polarized, and the positions of public health officials are met with skepticism by many vaccine-hesitant Americans. This polarization may impact future vaccination efforts as well as clinician-patient relationships.

**Methods:**

We interviewed 77 vaccine-hesitant patients and 41 clinicians about COVID-19 vaccination communication in primary care as part of a Veterans Affairs (VA) trial evaluating a vaccine-communication intervention. This paper reports the findings of a qualitative analysis focused on one aspect of those interviews—the disconnect between primary care clinicians’ and patients’ perceptions about COVID-19 vaccination communication and decision-making.

**Results:**

Rapid qualitative analysis of semi-structured interviews revealed fundamental differences in how clinicians and patients understood and described the reasoning, values, and concerns underlying COVID-19 vaccine hesitancy. These differences were significant and value-laden; they included negative judgments that could undermine communication between clinicians and patients and, over time, erode trust and empathy.

**Conclusion:**

We advocate for empathic listening and suggest communication strategies to bridge the divide between clinicians and vaccine-hesitant patients.

**Supplementary Information:**

The online version contains supplementary material available at 10.1186/s12875-024-02665-1.

## Background

In the United States (US), the COVID-19 pandemic era may be remembered, in part, for deepening fissures in trust between the public and public health experts and, perhaps also, between patients and the health systems that serve them [1–3]. In the US, mandates and social penalties bolstered efforts to vaccinate widely against COVID-19—efforts that were accompanied by other controversial pandemic control measures such as restrictions on social interactions and business operations [4]. A stark divide, mirroring the right-left political divide, came to characterize much discourse on vaccination, leaving the expert opinions of public health officials at odds with the views of many Americans [5–8]. It remains unclear what impact this era of polarization will have on vaccination efforts going forward, how it may impact clinician-patient relationships, and how to move forward.

In this paper, we explore how pandemic-era divisions are both reflected in and perpetuated by patient and clinician perspectives on COVID-19 vaccination. We do so by reporting the results of a focused qualitative analysis of veteran patient and clinician interviews conducted for a VA-based clinical trial evaluating an educational intervention designed to help clinicians discuss COVID-19 vaccination with their veteran patients [9]. The aim of this analysis was to map out discrepancies in how vaccine-hesitant patients and vaccine-promoting clinicians described the concerns, values, and reasons underlying vaccine hesitancy. A secondary aim was to explore the potential impact of these discrepancies on vaccine-related communication and on patient-clinician relationships.

Healthcare clinicians’ recommendation to vaccinate is strongly correlated with patients’ acceptance of vaccination [10–12]. Yet *how* clinicians interact with vaccine-hesitant patients may be more impactful than the content of their messages [11, 13, 14]. Recent literature on vaccine hesitancy recommends best practices for improving vaccination rates, including the use of presumptive language (language that normalizes vaccination by presuming intent to vaccinate), tailored messaging (vaccine communication that is specific to the individual or the communities to which the individual belongs), and personal testimonials (clinicians sharing their own experiences and personal reasons for endorsing vaccination) [15–19]. Motivational interviewing techniques, which elicit patients’ values, embrace open-ended inquiry, and emphasize patient autonomy in health decision-making may be especially promising for vaccine communication [20–22]. By contrast, attempting to dispel myths, fill presumed knowledge gaps, or provoke fear about the consequences of declining vaccination can be counterproductive [15–19].

Current literature also acknowledges that the patient-clinician relationship can have a significant impact on conversations and decisions about vaccination [23–26]. Without a foundation of trust and mutual respect, it is difficult for patients and healthcare clinicians to communicate meaningfully and effectively [1, 27]. How to foster trust in clinical relationships remains an understudied topic [28], but research suggests that “openness, honesty, and a willingness to admit errors and correct them…. are qualities all providers can use to create, build, and sustain trust” [29]. It is important that patients not only respect their clinicians’ skill, knowledge, and integrity, but also that they believe their clinicians, in turn, understand and respect them. As Khullar, Darien, and Ness [3] have written, “Patients want to be seen… as complex people engaged in healing, bidirectional relationships with their care teams.”

The present study adds to existing literature by examining perception gaps between vaccine-hesitant patients and vaccine-promoting clinicians as a potential obstacle to trust and mutual respect. Here, “perception gaps” refer to significant differences in perspective or belief related to patients’ vaccination decisions and their reasons for those decisions. In this paper, we characterize these gaps using qualitative interview findings, and we explore how they may impede meaningful engagement between patients and clinicians on controversial or sensitive health topics like COVID-19 vaccination. In describing these perception gaps, our purpose is not to identify myths or to fact-check providers or patients; rather, we seek to understand where significant differences in perspective exist and how they might become obstacles to meaningful communication and mutual understanding. We conclude by examining the implications of our findings for patient-clinician communication and relationships in the post-pandemic era.

## Methods

This paper presents the results of a secondary analysis of semi-structured qualitative interview data collected for a large, VA-based pragmatic implementation-effectiveness trial evaluating a virtual, motivational-interviewing-informed training designed to help clinicians increase COVID-19 vaccine acceptance rates among their patients [9]. During the post-intervention phase of the parent study between October 2022 and August 2023, 77 veteran patient interviews and 41 healthcare clinician interviews were conducted. These examined a wide range of factors affecting vaccination conversations, patient vaccination decisions, access to vaccination, and implementation of the study intervention. During rapid analysis of interview findings, analysts noticed that how providers’ perceived vaccine-hesitant patients’ values and decisions was often very different from how those patients viewed themselves. We therefore undertook the focused secondary analysis that is the topic of this paper, with the aim of addressing a new research question: In what ways do patients and clinicians differ in how they perceive patient-provider communications, interactions, and decision-making related to COVID-19 vaccination? To address this question, analysts reviewed content across all interviews and all domains in order to systematically identify, describe, and characterize key differences.

Patients were recruited from 10 different VA medical centers and their affiliated outpatient clinics across urban and rural settings in 7 states across the Southern and Western United States. They were selected for interviews using purposive sampling to ensure inclusion of diverse participants within prespecified categories (vaccination status, study site, geographical region, gender, age, race/ethnicity). At the time of their interview, 24 patients were unvaccinated; 53 were vaccinated but had been hesitant or had significantly delayed vaccination (i.e., were vaccinated 10 months or more after COVID-19 vaccines were available to the general population). Demographics for participating patients are presented in Table 1.

**Table 1:** Patient participants (*n* = 77)

COVID-19 Vaccination Status	Count
• Vaccinated	53
• Unvaccinated	24
**Race**	
• White	42
• Black	16
• Multiracial	10
• Asian/Pacific Islander	3
• Native American	2
• Unknown or Declined	4
**Ethnicity**	
• Not Hispanic or Latino	67
• Hispanic or Latino	8
• Unknown or Declined	2
**Gender Identity**	
• Man	50
• Woman	27
• Other	0
**Age**	
• Under 50	44
• 50 and Over	33

Clinicians were recruited from the same clinics and medical centers as patient participants. The clinician sample was assembled through a combination of random sampling and network sampling; invitations were sent in small batches to randomly-ordered lists of primary care clinicians at each site, and each interviewee was asked to recommend other potential interviewees involved in primary care and vaccination efforts at their site; site-level study collaborators were also invited to recommend clinician interviewees. Interviewed healthcare clinicians included registered nurses, licensed practical nurses, nurse practitioners, physicians, pharmacists, and a psychologist, all of whom worked in primary care clinics and/or were involved in their facility’s official vaccination effort (Table 2).

**Table 2:** Healthcare Clinician participants (*n* = 41)

Healthcare Clinician Occupation	Count
Registered Nurse	13
Licensed Practical Nurse	10
Nurse Practitioner	8
Physician	7
Pharmacist	2
Psychologist	1

Interviews covered a wide range of topics related to COVID-19 vaccination and were guided by semi-structured instruments developed for the parent study. Patient interviews included questions assessing the following topical domains: reasons for delaying/declining COVID-19 vaccine, prior experiences with COVID-19, experience discussing the COVID-19 vaccine with VA clinicians, trust/distrust in sources of vaccine information, contributors to perspective change (if applicable), reasons for getting vaccinated (if applicable), access to/process of getting the vaccine (if applicable), and current feelings about the decision. Clinician interviews included questions assessing the following domains: challenges in getting patients vaccinated, reflections on COVID-19 vaccine promotion efforts (what worked and what didn’t), experience using motivational interviewing to promote vaccination, and recommendations for how to encourage patients to receive COVID-19 vaccines. Interviews lasted 30 min on average and were audio-recorded for analysis.

Rapid Analysis Procedures [30–32] were used to develop a summary of content for each audio-recorded interview. This analytic approach was developed for structured, rapid-turnaround health services research projects like this one; it is time- and resource-efficient, yielding results comparable to traditional qualitative methods [30, 33, 34]. Guided by interview audio-recordings, analysts prepared a structured summary of each interview using a spreadsheet-based template organized by content domains drawn from the interview guide. To populate each template, the analyst briefly summarized interview content for each template domain and added relevant participant quotations alongside each summary. To ensure consistency among analysts, at least 20% of summary templates were reviewed by a second analyst, and any discrepancies were noted in writing and discussed. Throughout this process, the research team met to discuss preliminary themes, questions, and any process issues at weekly meetings devoted to qualitative analysis.

For the focused secondary analysis that is the topic of this paper, analysts reviewed summary template content across all interviews and all domains and systematically mapped out differences between the patient and clinician interviews in a separate Excel-based analytic matrix with columns dedicated to: topical domain, preliminary theme title, description of theme and relevant context, citations of all relevant interviews, citations of all exceptions and counter-examples, and relevant participant quotations. Theme identification was an iterative, collaborative process. Informed by weekly team discussions, individual team members reviewed matrix content and added drafts of preliminary themes in each domain between team meetings. Team members then worked together during research team meetings to discuss, organize, and condense matrix content and, ultimately, to fully describe and refine themes within each domain.

## Results

Interviews revealed fundamental differences between clinicians’ perceptions of vaccine hesitancy and vaccine-hesitant patients’ self-perceptions and stated rationale for their vaccine decisions. Clinician perceptions sometimes included attribution of undesirable motives and characteristics to vaccine-hesitant patients. Here, we describe five key perception gaps that could impact patient-clinician communication and relationships. These gaps are summarized in Table 3.

**Table 3:** Clinician-patient perception gaps related to Vaccine Hesitancy

Perception Gap Area	Clinicians tended to believe…	Patients tended to believe…
The Quality and Availability of Vaccine Information	High-quality information on COVID-19 vaccine safety and effectiveness is available.	Significant questions about the COVID-19 vaccines remain unanswered.
Reasons for Vaccine Safety Concerns	Misinformation, propaganda, and conspiratorial thinking are primary drivers of vaccine hesitance.	Their own prior experiences and the experiences of loved ones created concerns about vaccine safety and effectiveness.
Emotion versus Reason	Vaccine concerns are primarily rooted in politics and emotions.	There may be rational grounds for doubt and mistrust of official information.
Faith in Science	Vaccine-hesitant patients lack basic scientific literacy or reject science.	Being well-informed and advocating for unbiased science requires critically evaluating available vaccine information.
Personal Health and Public Health	Vaccine hesitancy stems from a failure to understand personal risk or to consider public-health implications.	A desire to protect themselves and the vulnerable underlies skepticism of the public-health rationale for vaccination.

Perception Gap 1: The Quality and Availability of Vaccine Information.

- Clinicians feel that high-quality information on COVID-19 vaccine safety and effectiveness is available.
- Vaccine-hesitant patients feel that basic questions about the COVID-19 vaccines remain unanswered.

The clinicians we interviewed generally expressed confidence that clear, high-quality information about COVID-19 vaccines is available and felt capable of educating patients adequately about vaccination. They repeatedly and confidently referenced “the facts,” “the research” and “the evidence-based science that’s come out.” Interviewed clinicians tended to believe that, if their patients were open to learning about COVID-19 vaccines, they could provide clear and relatively unambiguous evidence in support of COVID-19 vaccination for virtually everyone.

The vaccine-hesitant patients we spoke with had a very different perception about the availability of vaccine information. They described a dearth of facts addressing their specific questions about vaccine safety—including how vaccination might impact their own autoimmune conditions or other chronic health conditions. Explained one patient, “My biggest hang up was trying to… figure out what the different side effects are, because I suffer from [a chronic medical condition] and so I can’t just take certain things, so I have to make sure it’s going to be okay… Your everyday person doesn’t have to deal with that. So that person having the shot versus me having the shot may have different outcomes.” Patients observed that, because the vaccines are newer, limited information is available to describe how they may impact specific patient populations, and no studies have evaluated potential long-term impacts of COVID-19 vaccination. This was a major concern for the patients we interviewed: “Nobody could legitimately educate anybody else because nobody knew anything about it. [The COVID-19 vaccines] hadn’t been out on the market long enough. " Patients also noted that, because the virus is ever-evolving, study results and data about effectiveness can become obsolete before they are even released. This can complicate risk-benefit analyses.

When clinicians shared information about vaccination, patients felt it was often simplified and one-sided. They noted that their clinicians rarely acknowledged or discussed known side effects of COVID-19 vaccines because they were invested in encouraging vaccination:

“They weren’t very forthcoming [about potential side effects] or they would act like they didn’t know or they’d act like they didn’t want to scare me. It was more of a ‘oh, you’re better off getting it’…. The positives were far more of the conversation than what I was asking. I wasn’t getting the specifics on the bad, but I was getting more specifics on the good.”

Focusing on benefits without acknowledging and discussing risks did not inspire confidence among vaccine-hesitant patients.

In short, patients described significant gaps in the information they received about COVID-19 vaccines. When they asked their healthcare clinicians questions about the vaccines, they rarely received satisfying answers (or, in some cases, any answers). For example, one patient described asking his clinician “lots” of questions and “I couldn’t get any straight answers and so that’s what… was so frustrating and why I didn’t get it… Because I was getting the runaround.” Patients found that clinicians were often unable to provide requested information, such as lists of vaccine ingredients. This led to frustration and reinforced hesitance: “I’m not going to take something if I don’t know what’s in it.” One patient reasoned that his healthcare clinicians “are probably giving me scientific and medical reasoning. I don’t think they are lying to me. I just don’t think they know enough about it. That’s it, plain and simple.”

Perception Gap 2: Reasons for Vaccine Safety Concerns.

- Clinicians felt that misinformation, propaganda, and conspiratorial thinking are the primary drivers of vaccine hesitance.
- Vaccine-hesitant patients expressed concerns about vaccine safety that were often related to their own prior observations and experiences or the experiences of loved ones.

Clinicians overwhelmingly felt that patients’ safety concerns were rarely reality-based or fact-based. Instead, they were driven by misinformation and “conspiracy theories”—for example, the idea that the vaccines contain a tracking device. Clinicians felt that the concerns of many vaccine-hesitant patients were rooted in inaccurate beliefs and sometimes dismissed them as “tin-foil hat stuff,” “fake narratives,” and “silly conspiracies.” Clinicians concurred that, more than anything else, “false information is really detrimental and has influenced veterans in choosing not to get that vaccine.”

Patient interviews did sometimes reveal inaccurate beliefs and misunderstandings about how vaccines work. However, patients’ concerns were overwhelmingly tied to a perception that the vaccines were rushed into production rather than any specific theory about harm or any notion of conspiracy: “Vaccines usually take 5–10 years to be approved and the COVID vaccine was approved in less than a year? " Patients voiced particular concern about the relative novelty of mRNA technology for vaccination and noted the lack of long-term clinical trial data for this type of vaccine: “There was not enough study done on it to know what’s the long-term results.” Patients mentioned other drugs that were approved and later discontinued; they worried that, many years hence, scientists might recognize negative side effects that are currently only anecdotal:

We know we can look back in time and see other [similar] scenarios…. it comes back ‘oh, that was a bad idea, this causes cancer’…. Are we going to look back in time and find out that they were wrong about this vaccine, however it was produced, and be in a worse situation than I feel like [we] could have been?

Interviewed patients cited their own firsthand experiences and the experiences of their loved ones far more often than generic conspiracy theories as grounds for their concerns about vaccine safety and effectiveness. Explained one patient:

Having worked in the regulatory side of drug research, it [the COVID-19 vaccine approval timeline] seems fairly swift to me. So, I definitely have concerns. And I have known family members to have had adverse reactions to them [COVID-19 vaccines], but I also know that that is common for any type of treatment. I just have concerns about how swiftly it was tested and implemented.

Seeing vaccinated loved ones have difficult experiences with COVID-19 after vaccination or seeing them struggle with vaccine side effects was a significant factor in vaccine hesitance: “Everybody that I know that got the shot got sick [got] way sicker than I did” from COVID-19.

Additionally, multiple patients reported negative experiences related to receiving involuntary vaccines during their military service, sometimes with severe or lasting side effects that created ongoing concerns about vaccine safety. Shared one veteran, “I’ve had shots in the day… through the military that made me extremely sick…. The last time I let you guys dope me up with a bunch of vaccines, it nearly killed me. Why am I gonna come in here now and have you just shoot something into me and I have no clue what it is?”

Perception Gap 3: Emotion versus Reason.

- Clinicians see patients’ vaccine concerns as primarily rooted in politics and emotions.
- Vaccine-hesitant patients see rational grounds for doubt and mistrust of official information.

Clinicians tended to see vaccine hesitancy as rooted in politics and emotion rather than facts or reason. They noted that conservative politics and vaccine hesitance would often go hand in hand; for this reason, refusing the COVID-19 vaccine could serve as a way for patients to express their conservative identities: “The big issue that we face here was that it [COVID-19 vaccination] was so politicized that those that didn’t want it, felt it was, you know, the American thing to do to turn down the vaccine.” As such, clinicians saw patients as politically and emotionally invested in declining vaccination. In the words of one clinician, COVID-19 vaccination “has become so politicized that… it becomes an emotional issue rather than something that’s rational—rationally evaluated.”

Clinicians also described unvaccinated patients as distrustful of the government and authorities: “There’s just a mistrust, a general mistrust with the whole thing.” These patients were seen as stubborn and “very, very stuck in their ways”: “It’s mentality. Sometimes, people are set in their ways and they have beliefs, and these beliefs are unbreakable.” When clinicians perceived the concerns of their patients to be irrational, they doubted that those patients would be open to information or education about vaccination: “They already have their mind made up anyway.”

Patients did indeed voice distrust in official information about vaccination, and some described an alignment between their conservative politics and their decision to decline vaccination. However, these patients felt that there are legitimate and rational grounds to mistrust major sources of information about vaccines. Distrust of the news media was widespread among interviewees, with patients citing examples of news channels with perceived bias. Shared one patient, “It’s harder to believe what you hear nowadays, even on the news, because, to me, everything just sounds opinion-based now.” Another stated, “I’ve lost faith in them [the news media]. They even have their own dog in the fight… who they feel they’re behind, and that’s what the news is normally skewed towards.”

Patients also endorsed distrust of pharmaceutical companies and other entities involved in marketing the vaccine. Their distrust was grounded in how those companies stand to profit from vaccines, as well as the history of pharmaceutical companies profiting from harmful products. One patient described a lack of trust in “anybody making money from it [the vaccine], anybody with an invested interest in it—because if it’s great they’re going to tell you it’s great but if it’s terrible they’re also going to tell you it’s great.”

The patients who described distrust of federal government authorities (e.g., the CDC and the FDA) often directly linked this distrust to their own military experiences, which, for some, engendered lack of confidence and even feelings of betrayal. One veteran recalled, “I was in the Air Force. Every time we went somewhere, I was getting shot up with something and I didn’t know what it was… I don’t even know if they were FDA approved because the military and the Veterans Administration, they seem to get around some of that stuff.” When considering vaccination, another patient “had a flashback of my military days” and concluded, “I just don’t trust the United States government.”

Vaccine-hesitant patients were much less likely to express distrust of their VA medical clinicians. They generally trusted them and valued their opinion. Most suggested that additional discussion with trusted medical professionals could influence their decision about vaccination. However, some patients also lacked faith in the healthcare system and pointed toward perceived ways that it has been skewed by profit motives, and how those motives can jeopardize patient care and wellbeing. One patient questioned why he should trust a clinician he has no real relationship with: “I know healthcare providers are supposed to have your best interests out, and the whole Hippocratic oath and all that kind of stuff, but someone who only sees you three or four times out of 365 days of the year—I mean, why would you think they have your best interests at heart?”

In short, although patients and clinicians were aligned in citing distrust of selected authorities and information sources as a factor in vaccine hesitance, their perceptions diverged sharply regarding whether this distrust was warranted or rational. In contrast to interviewed patients, very few interviewed clinicians suggested that there might be some grounds to mistrust information shared by the mainstream news media, pharmaceutical companies, or federal authorities.

Perception Gap 4: Faith in Science.

- Clinicians see vaccine-hesitant patients as rejecting science and/or lacking basic scientific literacy.
- Vaccine-hesitant patients see themselves as informed and as advocates of true, unbiased science.

Clinicians described vaccine-hesitant patients as either rejecting science or lacking the literacy and comprehension skills necessarily to interpret scientific literature. Underlying vaccine hesitance was a “lack of knowledge”: “If you’re not informed and you honestly don’t understand the process—the disease process—then you’re not going to get [vaccinated].” Vaccine-hesitant patients were often described as gullible consumers who believe what they read on the internet and are neither critical nor discerning. For example:

With the advent of social media, a lot of times, what they will see on some of the social media networks and channels and things like that, they’ll come back saying ‘well, I saw on the news that it’s not really effective,’ or ‘I saw that this person had this bad outcome, so I’m definitely not going to get the shot.’ So those are kind of the things, unfortunately, we have difficulty controlling because that’s all out there in social media.

Another clinician joked that, to address vaccine hesitancy, “we need to cut off all of their news channels and their access to the internet.”

When clinicians saw patients as lacking understanding of or respect for science, they concluded that additional data would probably not influence patients’ decisions or change their minds about vaccination. For example, one clinician described vaccine-hesitant patients as “unwilling to listen to science versus whatever they’ve heard from Neighbor Joe down the street.” When asked what might make a difference to these patients, some clinicians made resigned comments such as: “I don’t know what it would take to change their mind, honestly. Because they are so committed to that side of it, their side.”

Many vaccine-hesitant patients had an almost diametrically opposed self-perception; they saw themselves as critical consumers of information and advocates of true, unbiased science. Patients described the importance of examining information sources with a critical eye and believed that they were careful, discerning consumers. When asked what information sources they trusted, they often touted the importance of “peer-reviewed” research, “double-blind stud[ies],” and the “scientific method” and many noted that you cannot trust much of what you read on social media or the internet.

Patients consistently emphasized the need for large-scale, long-term research to understand the safety and effectiveness of new vaccines, and frequently attributed their hesitance to lack of adequate data: “There wasn’t enough data that I thought was available to the public to make a well-informed decision about whether or not it was fully safe.” Often, patients’ complaints about the vaccine roll-out and vaccine promotion efforts were grounded in a sense that these were “not scientific” or didn’t represent “good science.” They worried that the approval process for vaccination was tainted by politics. With some exceptions, patients suggested that stronger, longer-term data could influence their decisions. In short, like clinicians, patients overwhelmingly considered themselves to be believers in high-quality, unbiased science.

Perception Gap 5: Personal Health and Public Health.

- Clinicians believe vaccine hesitancy is tied to patients’ failure to understand their personal risk/vulnerability or to consider public-health implications.
- Vaccine-hesitant patients are deeply skeptical of the public-health rationale for vaccination and may believe they are protecting others as well as themselves by choosing not to get vaccinated.

Clinicians often felt that the best way to appeal to vaccine-hesitant patients was to confront them with their own risk of severe disease or death from COVID-19: “You kind of put it in their minds that the COVID [virus] is still out here and that they do have health issues or chronic health concerns that will put them at a higher risk of having a severe infection if they are infected. It kind of maybe changes their perspective a little bit because they know that they are at a higher risk.” Implicit in this strategy was an unspoken notion that vaccine-hesitant patients may not be motivated by public health considerations and/or may make vaccination decisions based solely on personal health considerations. Clinicians noted that vaccine-hesitant patients tend to underestimate their own risk of complications from COVID-19—for example, they may think they are healthy and strong so they don’t need to be vaccinated. Or, in some cases, they may just be just tired of hearing about COVID-19 and don’t think it is really relevant to them. As such, something may have to “scare them” into choosing to get vaccinated: “We go for a fear factor kind of thing. We base it on mortality, we base it on statistics…. We give them examples.”

However, patients who chose not to get vaccinated did not necessarily refrain because they felt that they themselves were healthy, strong, and at lower risk for COVID-19. While some felt this way, others described themselves as particularly vulnerable to severe disease. These included patients who had autoimmune conditions, heart conditions, prior cancer diagnoses, and even prior hospitalizations with COVID-19. Many had lost friends or family members to COVID-19. However, they remained concerned that, for them, and for other vulnerable populations, vaccination could be worse than COVID-19 disease. Explained one patient:

I do not believe the vaccine is safe… [M]y brother-in-laws, they all got the shot and they’re dead now. My sister-in-law, she got the shot and she’s dead and she got the COVID after she got the shot. And my father-in-law, same thing—he got the shot and died and he was healthy as a big dog. He walked miles every day, he swam miles and he got the vaccine and then three days later her got sick and two days later he was dead.

Research may demonstrate that vaccination saves lives, but anecdotes like this one reflect powerful personal experiences that had an indelible impact and undermined faith in the public-health rationale for vaccination.

In short, vaccine-hesitant patients were not exactly dismissive of public health considerations related to vaccination, and their decisions were not based exclusively on an assessment of their personal vulnerability to COVID-19. Instead, these patients were deeply skeptical of the public-health rationale for getting vaccinated against COVID-19. By not getting vaccinated, they felt they were protecting themselves. By sharing their concerns about the safety and effectiveness of the vaccine, they felt they were protecting others, including vulnerable friends and family members.

## Discussion

The purpose of this paper is not to adjudicate the relative merits of vaccine-hesitant patients’ perceptions or their clinicians’ perceptions. We readily acknowledge that current scientific literature supports the position that the COVID-19 vaccines available in the United States are safe for the vast majority of people and are effective at reducing risk of hospitalization and death [35–37]. Our point, instead, is that there is a mutual “mis-seeing” that may not only impede conversations about vaccines but may also affect patient-clinician relationships in other ways. We found that the motivations and values that clinicians attribute to vaccine-hesitant patients may sometimes be very different from patients’ stated motivations and values. Further, clinicians may underestimate patients’ openness to and interest in discussing vaccination. We suggest that a deeper understanding of vaccine-hesitant patients’ values and perspectives may be needed to improve communication about sensitive topics like vaccination and to prevent erosion of trust in the patient-clinician relationship. Our interview findings suggest several ways that healthcare clinicians can foster this deeper understanding and promote better communication.

Above all, our findings point to the need for better listening. We learned that clinicians and patients do not simply disagree regarding the merits of vaccines; clinicians may sometimes fundamentally misunderstand patients’ concerns and perspectives. Interviews showed that clinicians can be reluctant to broach the topic of COVID-19 vaccination with hesitant patients. This may stem, in part, from a not-always-accurate belief that vaccine-hesitant patients are uninterested in learning more about vaccination. Unlike our interviewers, busy healthcare clinicians do not have the luxury of an uninterrupted 30-minute conversation with patients about vaccination. But even much shorter conversations may make a difference over time if they involve genuine listening and help to soften the underlying assumptions of both patients and clinicians [38, 39]—for example, patient assumptions that clinicians cannot address their concerns or clinician assumptions that unvaccinated patients are too set in their ways to consider new information.

A listening-centered approach, consistent with motivational interviewing, allows patients to guide discussions and focus the conversation on the aspects of vaccination that matter the most to them. MI is a collaborative technique that uses open-ended questions to elicit the patients’ values and concerns, conveys respect for patient autonomy and decision-making, and offers information collaboratively with patient permission [20, 21]. MI has a strong evidence-base across a variety of targeted health behaviors in medical settings (e.g., smoking cessation, treatment engagement, dietary changes) and has been identified as a promising intervention to address vaccine hesitancy [20–22, 40]. If clinicians invite patients both to share their values and to voice their questions and concerns about vaccination, clinicians might be less likely to attribute characteristics like ignorance, gullibility, stubbornness, and irrationality to vaccinate-hesitant patients—implicit attributions that could have an enduring negative impact on the patient-clinician relationship.

Our interviews suggest that hearing, acknowledging, and validating patients’ prior negative experiences with vaccination may be one way to build trust and prevent damage to the patient-clinician relationship. Patients readily shared vaccine-related anecdotes based on their own experiences and the experiences of loved ones; these experiences were sometimes disturbing or even traumatic and were seldom acknowledged or recognized in healthcare conversations about vaccination. Listening to these experiences could be a powerful act that builds empathy and fosters mutual trust. It also opens the door for clinicians to tailor their messaging to patients’ needs and even to share personal stories about their own experiences and observations—communication strategies that can foster mutual understanding [41] and that show promise in recent literature on vaccine hesitancy [15, 17].

It is similarly important to acknowledge and contextualize potential risks and complications of vaccination, even if they are rare. Patients wanted to hear from their clinicians about vaccine risks; they wanted to be informed and to make educated decisions. Hearing only a “sales pitch” for vaccination (all the positives without potential negatives) did not inspire confidence. Patients wished that their clinicians would be forthright about what they (and the scientific community) do not yet know as well as what they do. Montgomery, Berns, and Braddock [41] identified this sort of transparency as prerequisite to building a culture of trust in healthcare in the wake of the COVID-19 pandemic. When clinicians embrace humility and acknowledge medical fallibility, it can humanize them and help build the trust necessary for effective communication about sensitive topics like vaccination.

Our findings point toward trust in science as an area of potential common ground that may facilitate communication. Vaccine-hesitant patients, like their clinicians, often do care about science. Patients, like clinicians, are generally committed to understanding and addressing bias. Patients, like clinicians, emphasize the importance of consuming information carefully and critically. These are shared values that clinicians can draw on when discussing vaccination with patients. The vaccine-hesitant patients we interviewed generally wanted more in-depth discussion about vaccine research, not less. They were seldom satisfied with simple fact sheets presenting generic information about vaccines. Some were willing to dive into journal articles and wanted to be directed to relevant scientific literature; some had backgrounds in health professions themselves. But their clinicians sometimes assumed that they were ignorant, confused, and lacking in scientific literacy. As such, they failed to leverage potential common ground for communication.

Of course, despite both patients’ and clinicians’ desire for facts and faith in science, studies show that “vaccine knowledge does not predict vaccine hesitancy and educational interventions have little to no impact on trust in vaccine” [15]. This supports the theory that *how* clinicians communicate with patients may be at least as important as *what* information they share [13, 14]. By embracing communication strategies that are grounded in empathic listening and mutual respect, such as motivational interviewing [38, 42], clinicians are more likely to create the foundations for effective communication and a trusting relationship. This has implications that extend far beyond conversations about COVID-19 vaccination.

Our recommendations to improve patient-clinician communication align closely with the promising practices identified in a mixed-methods study conducted by Zulman and colleagues in 2020 [43]. Combining a systematic literature review, direct observation of healthcare encounters, qualitative interviews with patients and clinicians, and a Delphi panel process, they identified several strategies to promote “presence and connection with patients in the clinical encounter.” Among them are listening “intently and completely” and learning “what matters most” to the patient so that the patient’s priorities can guide the encounter. Zulman and colleagues also note the importance of recognizing and validating emotional cues and “connect[ing]with the patient’s story,” which may include learning about how patients’ personal experiences have affected their health and their healthcare. Our interview findings suggest that these promising practices are not yet routine in vaccine-related interactions between healthcare clinicians and vaccine-hesitant patients. Adopting such practices in the challenging context of conversations about COVID-19 vaccination could promote connection and strengthen, rather than undermine, the patient-clinician relationship.

### Limitations and directions for future research

Veteran patients served by the VA are a unique population and their experiences may be different from those of other patients. It is thus not clear how well our findings generalize outside of veteran populations and VA settings, which have their own unique and specific culture, structure, and processes. In some cases, the vaccine-hesitant veterans we interviewed explicitly referenced their military experiences to explain their concerns about vaccination and their distrust of government information sources. The experiences of other patient and clinician populations may differ in significant ways and deserve research in their own right.

Further, even within the veteran population, vaccine-hesitant patients are not a monolithic group [44]. Although our qualitative sample was large and diverse, our study was not designed to compare and contrast the experiences of different veteran subgroups (for example, different gender, age, and racial or ethnic populations). Future research could examine how the experiences of different veteran subpopulations differ and could point toward more tailored approaches to improving patient-clinician communication and trust.

## Conclusion

Martin Luther King Jr. is said to have cautioned that, “you have very little morally persuasive power with people who can feel your underlying contempt” [45]. Contempt is almost certainly too strong a word for the implicit (and sometimes explicit) judgments we noticed in clinicians’ statements about vaccine-hesitant patients. But King’s message resonates nonetheless: If patients sense that clinicians see their concerns about vaccination as irrational or rooted in ignorance, are they likely to have productive conversations about vaccination? Are patients likely to hear and respect the recommendations shared by clinicians who doubt their rationality or intelligence?

John Parrish-Sprowl [14] has observed that clinician-patient communication about vaccination always “occurs in the context of a conversation, not as a message standing alone, unfettered by the context of interaction between the parties.” For this reason, he argues that “[t]he process of conversation is where vaccine hesitancy will be addressed, not in a singular message that stunningly persuades a person to shift to acceptance.” We argue further that these conversations occur in the context of a relationship—one that is shaped over time, perhaps by many brief conversations and, also, by the unique histories, experiences, and perceptions of both parties. Tending to that relationship means prioritizing listening, even in a time-constrained environment, and embracing the fundamental values of humility and empathy. In a post-pandemic society rife with intense political and social division, there is much about the context of patient-clinician interactions that neither party controls. But we still have the power to welcome each patient with respect, to hear what matters to them, and to embrace empathy across our differences.

## Electronic supplementary material

Below is the link to the electronic supplementary material.

Supplementary Material 1

Supplementary Material 2

Supplementary Material 3

## Data Availability

Upon request, a limited dataset may be created and shared by the authors pursuant to a Data Use Agreement appropriately limiting use of the dataset and prohibiting the recipient from identifying or re-identifying (or taking steps to identify or re-identify) any individual whose data are included in the dataset.
