# Supplementary material for: When clinicians and patients disagree on vaccination: what primary care clinicians can learn from COVID-19-vaccine-hesitant patients about communication, trust, and relationships in healthcare
Source: BMC Prim Care. 2024 Dec 5;25:412. doi: 10.1186/s12875-024-02665-1 (PMC11619658; doi:10.1186/s12875-024-02665-1)
Supplement: Supplementary file 1 — Supplementary Material 1 [file 12875_2024_2665_MOESM1_ESM.docx]

**Aim 2 Qualitative Interview for Veterans who Choose to not get the COVID-19 Vaccine**

We are conducting a study that is trying to understand why some Veterans are vaccinated against COVID-19 and why others are not vaccinated. Your responses to these questions will help us understand the reasons why Veterans choose to get the vaccine or not. Just a reminder we are audio recording today’s interview so please try to not say your name or any other information that would personally identify you.

Grand tour question: Tell me about why you are choosing to not get the COVID-19 vaccine?

Follow-up questions:

1. What was the most important reason for you to not get the COVID-19 vaccine? And why?
2. Did you or someone you care about have COVID-19?
   1. Did this influence your decision to not get the COVID-19 vaccine?
3. What are some other reasons why you did not want to get the vaccine?
   1. Are there specific people who influenced your decision to not get the vaccine?
   2. Are there specific information sources that influenced your decision to not get the vaccine?
   3. Any other reasons why you did not get the COVID-19 vaccine?
4. Is there anything that might make you more inclined to get the vaccinated?
5. Did anyone at the VA clinic talk with you about getting the COVID-19 vaccine?
6. [If Yes] Please tell me about the conversation? [Probe for what was said and who said it – try to ascertain the role of the person too – was it the provider, the nurse, an aide, a peer, social worker, etc. And, also the veteran’s feelings about/reaction to the conversation.]

[Probe: Did you feel your questions were answered and your concerns were addressed?]

1. Did you get a phone call from someone at the VA about the COVID-19 vaccine?
2. What information have you received from the VA healthcare system regarding the COVID-19 vaccine?
   - 1. What else would you like to have seen?
3. Which people or sources do you trust the most to give you accurate information about COVID-19 vaccination (e.g. its safety, whether it is a good idea for you to get it, etc.)?
4. Which ones do you not trust?
5. Do you typically get vaccines, like an annual flu shot? Why or why not? [Listen for feelings about safety, effectiveness, trust, military experiences, etc.]

*(Questions 8 and 9 below are optional probes, if relevant.)*

1. How would you describe your likelihood of being exposed to COVID-19?
   1. Did your likelihood of being exposed to COVID-19 factor into your decision to not get vaccinated?
2. How sick do you think you would get if you contracted the COVID-19 virus?
   1. [Probe: What pre-existing health conditions do you have (if any) that could make COVID-19 worse for you if you got it?]
   2. Did considering how sick you may get impact your decision to not get vaccinated?
3. What would you say to a veteran who is considering whether or not to get the COVID-19 vaccine?
4. What could the VA do to better support Veteran’s in their decision making about the COVID-19 vaccine?
5. Do you have any other thoughts regarding the COVID-19 vaccine that you would like to share with me today?

THANK YOU for taking the time to speak with us today. I’m going to stop the recording now.

**Aim 2 Qualitative Interview for Recent COVID-19 Vaccine Acceptors**

We are conducting a study that is trying to understand why some Veterans have been vaccinated against COVID-19 and others have not. Just a reminder we are audio recording today’s interview so please try not to say your name or any other information that would personally identify you.

**Grand tour question:** Please tell me about why you decided to get the COVID-19 vaccine?

Follow-up questions:

1. What was the most important reason for you to get the COVID-19 vaccine? And why?
2. Did you or someone you care about have COVID-19?
   1. Did this influence your decision to get the COVID-19 vaccine?

3. Did anyone at your VA clinic contribute to your decision to get vaccinated? If yes, please tell me about the conversation and who you spoke with. [Probe for what was said and who said it – try to ascertain the role of the person too – was it the provider, the nurse, an aide, a per, social worker, etc.]

a. What was most important to you about these discussions?

- 1. [Probe: Did you feel that your questions were answered and your concerns were addressed?]
  2. [Probe: Were these discussions more informational or did they encourage you to get the COVID-19 vaccination or did you feel it was a combination of both?
  3. Did you get a phone call from someone at the VA about the COVID-19 vaccine?

How important were discussions with VA clinic personnel compared to other factors in you deciding to get the vaccine?

4. Prior to getting the COVID-19 vaccine, what were the main reasons why you did not want to get the vaccine?

1. Were there specific people who influenced your initial decision to not get the vaccine?
2. Were there specific information sources that influenced your initial decision to not get the vaccine?
3. What if anything changed your thinking about getting the vaccine? [probe for details]
   1. Was there a specific person who influenced your decision to get vaccinated?
   2. Were there specific information sources that influenced your decision to get vaccinated?
4. Which people or sources do you trust the most to give you accurate information about COVID-19 vaccination (e.g. its safety, whether it is a good idea for you to get it, etc.)?
   1. Which sources do you not trust to give you accurate information about COVID-19 vaccination?
5. Once you decided to get the COVID-19 vaccine, did you have any problems actually getting the vaccine? [If yes, probe for what the problems were and how they were resolved.]
6. How do you feel about your decision to get the COVID-19 vaccine now?

*(Questions 9 and 10 below are optional probes, if relevant)*

1. How would you describe your likelihood of being exposed to COVID-19?
   - 1. Did your likelihood of being exposed to COVID-19 factor into your decision to get vaccinated or boosted? [Probe: if yes, did it impact vaccine or booster or both]
2. How sick do you think you would get if you contracted the COVID-19 virus?
   - 1. [Probe: What pre-existing health conditions do you have (if any) that could make COVID-19 worse for you if you got it?]
     2. Did considering how sick you may get impact your decision to get vaccinated or boosted? [Probe: if yes, did it impact vaccine or booster or both]
3. What would you say to a veteran who is considering whether or not to get the COVID-19 vaccine?
4. What could the VA do to better support Veteran’s in their decision making about the COVID-19 vaccine?
5. Do you have any other thoughts regarding the COVID-19 vaccine that you would like to share with me today?

THANK YOU for taking the time to speak with us today. I’m going to stop the recording now.
