# Supplementary material for: When clinicians and patients disagree on vaccination: what primary care clinicians can learn from COVID-19-vaccine-hesitant patients about communication, trust, and relationships in healthcare
Source: BMC Prim Care. 2024 Dec 5;25:412. doi: 10.1186/s12875-024-02665-1 (PMC11619658; doi:10.1186/s12875-024-02665-1)
Supplement: Supplementary file 2 — Supplementary Material 2 [file 12875_2024_2665_MOESM2_ESM.docx]

VA Staff, PACT and HBCs Mid-Study Interview Guide:

I’m contacting you as a member of the CoVAcS study team. The goal of our study is to increase vaccination among Veterans who are hesitant to get the COVID-19 vaccine or experiencing logistic barriers (temporal, geographic, financial) to vaccination. The purpose of our interview today is to learn more about what has worked and not worked at your VA when it comes to helping veterans make decisions about vaccination and helping clinicians and staff have conversations with veterans about vaccination. We’ll focus on your experience over the last six months.

We are audio recording today’s interview so we will avoid using your name or any other information that would personally identify you once the recorder is turned on.

Do you have any questions before we get started?

1. I want to start by describing a training offered by our study team to see if you remember whether you participated in this training at some point over the last six months:

The CoVAcS training focused on using motivational interviewing as a communication strategy to increase COVID-19 vaccine acceptance. The training was delivered to groups of health care providers and staff via Teams by CoVAcS study personnel and/or health behavior coordinators at your VA. The training included information about COVID-19 infection rates at your VAMC and surrounding CBOCs and demonstrations for how to use motivational interviewing to improve COVID-19 vaccine acceptance.

- 1. Thinking back over the last 6 months, did you participate in this training? If so, please tell me about your experience of the training, including what was helpful and what wasn’t.
  2. Did you participate in any other training to help you communicate with veterans about the COVID-19 vaccine? If so, please tell me about your experience of the training, including what was helpful and what wasn’t.

1. What has been your experience, if any, using motivational interviewing to increase Veterans’ willingness to be vaccinated against COVID-19?
   1. Is this experience similar or different for primary vaccines and booster doses?
2. What other activities are taking place at your facility to promote the COVID-19 vaccine or booster? Can you share any details about these activities?
   1. For activities the participant took part in: Can you share your experiences with [activity], including what worked well and what didn’t?
3. What are some of the ongoing challenges your site is facing in getting Veterans vaccinated against COVID-19?
4. In your opinion, what would be the most effective ways to encourage Veterans to receive a vaccine for COVID-19?
   1. Which, if any, of these activities are currently happening at your site that you know of?
   2. What would it take, in your opinion, to get these types of activities up and running at your side?

1. What could VA do to get more providers and staff trained in effective vaccine communication strategies?
2. What is your experience with Veterans’ willingness to receive a flu vaccine, in comparison to COVID-19 vaccines?
3. How could VA efforts to improve vaccine acceptance better address racial disparities and other demographic disparities?
4. Do you have any final thoughts or suggestions regarding acceptance of the COVID-19 vaccines and boosters that you would like to share?
