# Supplementary material for: When clinicians and patients disagree on vaccination: what primary care clinicians can learn from COVID-19-vaccine-hesitant patients about communication, trust, and relationships in healthcare
Source: BMC Prim Care. 2024 Dec 5;25:412. doi: 10.1186/s12875-024-02665-1 (PMC11619658; doi:10.1186/s12875-024-02665-1)
Supplement: Supplementary file 3 — Supplementary Material 3 [file 12875_2024_2665_MOESM3_ESM.pdf]

Interview Set (Veteran or Provider)	Theme Short Title	Theme Description / Narrative	Examples VETS- VISN 1 [21] PROVIDER	Examples VETS- VISN 2 [] PROVIDER	Key Quotes & "Stories", if applicable (with citation attached)<br><br>VISN 1	Key Quotes & "Stories", if applicable (with citation attached)<br><br>VISN 2
Veteran	1. Info Readily Available vs. Basic Questions Unanswered	INCLUDE: LACK OF INFORMATION AVAILABLE / UNANSWERED QUESTIONS<br><br>Basic questions about vaccine safety and effectiveness remain unanswered.<br><br>Veterans feel that there is a lack of information / data available to address their specific questions about vaccine safety-- including what the constituent ingredients are and how the vaccines might affect their existing health conditions (especially autoimmune conditions).<br><br>Veterans also acknowledged how the situation w/r/t COVID is ever evolving as the virus evolves, making data about effectiveness obsolete before it is even released.<br><br>In short, they emphasized that a lot is unknown and that, when they ask questions about vaccines, they do not get satisfying answers (or, in some cases, any answers) to address them.	Vaxxed:<br>1_34857;<br>1_41044;<br>1_19013;<br>1_26889;<br>1_37066;<br>1_42698;<br>1_54262;<br>1_14569<br><br>Unvaxxed:<br>1_33955;<br>1_33227	Vaxxed:<br>2_30213;<br>2_39421;<br>2_40575;<br>2_46145<br><br>Unvaxxed:<br>2_37980;<br>2_38460;<br>2_51084	1_34857: "Nobody could legitimately educate anybody else because nobody knew anything about it. It hadn't been out on the market long enough. They fast tracked it."[22:45]<br><br>1_26889 - Trusts the medical field more or less, just doesn't think they have enough info. / "They are probably giving me scientific and medical reasoning. I don't think they are lying to me. I just don't think they know enough about it. That's it, plain and simple."<br><br>1_19013: "It seemed like there was a lot of unknowns still about it so it was hard to trust that we know about the vaccination when we didn't even know what the actual disease was doing" [9:29]<br><br>1_33955: "I don't know what the ingredients are, they don't list the ingredients and how this affects your body… I've read that they actually put mercury in the shot and then, no they don't… I feel like I'm allergic to the ingredients" [3:07] / "They're not giving out information, don't you get it? There is no information.... nobody's given me any information about the shot, I haven't seen a list of the ingredients" [11:42]<br><br>1_37066: "I simply just didn't have enough information about it [the vaccines]" [0:40]<br><br>1_37066: "It was just a matter of waiting and seeing: waiting for the right information and seeing what outcomes people were having." [7:50]<br><br>1_37066: "My biggest hang up was trying to find out and figure out what the different side effects are, because I suffer from hypertension and so I can't just take certain things, so I have to make sure it's going to be okay." [4:20]... "I suffer from hypertension, so your everyday person doesn't have to deal with that. So that person	2_30213: "I sure did, lots of them. And, I couldn't get any straight answers and so, that's what was at the very beginning - that's what was so frustrating and why I didn't get it at the very beginning because I was getting like the run around and stuff."<br><br>2_37980: "…People need to have informed consent and across the board in the allopathic medical community. Doctors themselves are not as informed about vaccines as they should be so they cannot give patients enough information to have true informed consent."<br><br>2_38460: "I read up on the information and it was experimental but it didn't have the ingredients listed…with it being experimental not a good plan for me."<br><br>2_39421: [When asked if anyone at their VA clinic contributed to their decision to get vaccinated] "No, they were useless about it."<br><br>2_46145: He stated that he believes we do not have enough information regarding covid and the vaccines were rushed out "I just though Covid was like the flu, and it was made out to be bigger than what it was"<br><br>2_51084: "I'm not going to take something if I don't know what's in it."
Veteran	2. Conspiracy Theories vs. Legitimiate Grounds for Doubt and Mistrust	INCLUDE: REASONS / RATIONALE FOR DISTRUSTING OFFICIAL INFORMATION SOURCES<br><br>Veterans feel that there are legitimate grounds for doubt and mistrust of official information about vaccines, especially from mainsteam news sources.<br><br>Distrust of news media is the most common, with respondents citing bias.<br><br>Distrust of pharmaceutical companies and entities marketing the vaccine is grounded in how they stand to profit from vaccines, and the history of their profiting from harmful products.<br><br>Distrust of the government and the CDC may be tied to military experiences.<br><br>Distrust of providers and the medical establishment is less common; interviewees who were vaccine hesitant generally trusted their doctors. But some lacked faith in the healthcare system and pointed toward the ways that it has been skewed by profit motives, and how those can jeopardize patient	Vaxxed:<br>1_26889;<br>1_33918;<br>1_34857;<br>1_41044;<br>1_10758;<br>1_22420;<br>1_31899;<br>1_37066;<br>1_39502;<br>1_41550;<br>1_46356;<br>1_47362;<br>1_54262<br><br>Unvaxxed:<br>1_30477;<br>1_35025;<br>1_38082;<br>1_25537;<br>1_33227	Vaxxed:<br>2_33802;<br>2_35166;<br>2_35718;<br>2_38576;<br>2_45060;<br>2_46145<br><br>Unvaxxed:<br>2_9607;<br>2_18007;<br>2_31703;<br>2_37989;<br>2_21727;	1_26889 - distrusts agenda-driven media<br><br>1_33918- Distrusts news agencies on both sides- doesn't like how the extremes politicize everything. Named dislike of CNN and Fox. Said he listens to both but does his own research. Followed CDC flu statistics and COVID stats and found it weird that all the flu stuff disappeared a couple months after covid. Felt they were quick to blame everything on covid. "it put me off."<br><br>1_34857 - "It just seemed to be fear-driven. Nobody knew anything about it…. Not only did nobody know anything really about COVID, the virus itself, but they knew even less about the vaccine. So all this propaganda they were pushing out was just blatant lies." [7:00]<br><br>1_34857 - "The CDC and the FDA, these are supposed to be science-based. They're supposed to be for the health and well-being of the community, and I don't believe that they are. I absolutely believe that all their decisions and all their releases and all the information they're piping out to the public, I absolutely believe that it's all politically driven." [19:45]<br><br>1_34857 - "I was a government pawn long enough to know not to trust them. Not to believe anything they're saying." [21:30]<br><br>1_34857 - "That's what healthcare seems to be now. You get a guy that comes in, you want to get him on the hook, you give him a bunch of meds, you order a bunch of tests, you get paid. Well, you're not helping this person. You are sustaining, you are keeping this person sustained." [35:05<br><br>1_41044: "You can't even trust anything anymore because everybody's willing to lie to	2_9607: "They're just talking, saying that so they could win an election [followed by participant's laughter]. There's politics behind it too." "…then you get into the pharmaceutical part of it and most of your senators and congressmen have their hands in Pfizer and Moderna"<br><br>2_18007: "Some media, they really are bias, and that goes for both sides…I don't know what's true."<br><br>2_31703: "Anything thats tied to or known with a policital stance That kind of goes between both ways. Someone with a medical background but without a political bias, or a financial bias from company backings."<br><br>2_33802: "I really don't trust the sources. I just decided to get the vaccine because I got sick. I think there's an entity out there where create a crisis… Yeah that's why I didn't get it at first. Because I know that a crisis is created by those in high government. [5:45] … I figure that the crisis that was created was COVID. So they create a crisis and then bring a solution. And then we say 'oooh hooray' good old USA, good old American people'."<br><br>2_35166: "I think that's all just a hoax to get people to follow in line and to do things."<br><br>2_35718: He also expressed specific distrust in CNN and MSNBC and stated that they had a history of misinformation that made then untrustworthy.<br><br>2_21727: "cant trust the news at all… and the internet is, you know, it's [inaudible] can't trust much of anything."

Veteran	3. Vaccine Safety Concerns Irrational vs. Rational	INCLUDE: CONCERNS ABOUT SAFETY AND REASONS FOR THOSE CONCERNS<br><br>Vaccine concerns are rooted in safety and managing risks to health and wellbeing.<br><br>Veterans' safety concerns about the vaccine are tied to the perception that the vaccines were rushed into production.<br><br>There is lack of long-term data and lack of reckoning with side effects.<br><br>Often, veterans' family members / loved ones had firsthand experience of negative side effects from the COVID vaccine that contributed their concerns and hesitance.<br><br>Veterans themselves reported firsthand negative experiences of receiving involuntary vaccines during their military service, sometimes with severe and lasting side effects that created ongoing concerns about the safety of new vaccines.	Vaxed:<br>1_26889;<br>1_33918;<br>1_34857;<br>1_41044;<br>1_10758;<br>1_14569;<br>1_17897;<br>1_28235;<br>1_31899;<br>1_37066;<br>1_37506;<br>1_37615;<br>1_39502;<br>1_41550;<br>1_42698;<br>1_43505;<br>1_43623;<br>1_46356;<br>1_47362;<br>1_14569<br>1_23226<br><br>Unvaxxed:<br>1_30068;<br>1_30477;<br>1_33955;<br>1_35025;	Vaxxed:<br>2_35718;<br>2_40978;<br>2_33452;<br>2_36531;<br>2_38576;<br>2_39421;<br>2_39620;<br>2_40575;<br>2_44705;<br>2_45060;<br>2_46145<br><br>Unvaxxed:<br>2_9607;<br>2_18007;<br>2_31703;<br>2_37989;<br>2_38516;<br>2_2804;<br>2_21708;<br>2_21727;<br>2_31733;<br>2_37980;<br>2_38004<br>2_38460;<br>2_46883	1_34857: "My resting heart rate is normally around 60... and now it's always elevated. Always. A couple weeks ago, I wasn't doing anything. I was just sitting on the couch and I hadn't gone anywhere, I hadn't gotten up and I had been sitting for quite a while. My resting heart rate was 113.... Nothing else in my life has changed except for that [the vaccine]." [29:00]<br><br>1_34857: "I wasn't immunocompromised before and I feel like I am now" [27:25]<br><br>1_41044: "I have rheumatoid arthritis as well, and so... I'm allergic to so much stuff and have so many issues with cross medications."<br><br>1_41044: "They were having all those people in the hospital from the vaccine have those heart problems…. Having heart palpitations and everything as soon as they were given the vaccine. So I was like 'Oh, great. And with all the problems that I have from taking certain medications, I don't need another problem." [15:10]<br><br>1_41044: "I just think it's incredibly fast for there to have been human trials for a vaccine for something that just came out this quick" [4:35]<br><br>1_10758: "We know we can look back in time and see other scenarios, or situations we were forced through, and then it comes back 'oh, that was a bad idea, this causes cancer'…. Are we going to look back in time and find out that they were wrong about this vaccine, however it was produced, and be in a worse situation than I feel like I could have been?" [8:10]<br><br>1_14569: "You don't know the long-term effects."<br><br>1_17897: Regarding safety of the vaccines: "I have concerns. Having worked in the	2_9607: "I just don't believe, I just don't believe it's been researched enough.I think it came out way too fast, and I think they'll probably be lawyers {inaudible: involved] in 10 years from now you're probably going to have somebody on TV wanting to get money because of the Covid vaccines. I just don't think they know enough about what they're doing and what this is."<br><br>2_18007: "I didn't trust it" "Possible health risks, vaccine can cause blood clots, myocarditis, or at least if you're a woman."<br><br>2_31703: "The biggest thing was the long-term study or clinicals that were done. I just don't think feel like they were long enough to know any long term effects"<br><br>2_35718: "I didn't think I needed it. I'm not at High risk … and there wasn't a lot of information about the long term effects"<br><br>2_37989: "Came out too soon. I had too many questions about it. Some of the contents of the vaccine were not good."<br><br>2_38516: "I think that they come out with [the Covid vaccine] too quickly and I did work in the medical field so I'm leery of things that come out too quickly."<br><br>2_2804: "They come up with the vaccine really fast and I don't trust it."<br><br>2_21708: "They are pushing it through for everyone to get it, and it hasn't been tested all that much with the Food and Drug Administration."<br><br>2_21727: "There was not enough study done on it to know whats the long term results."
Veteran	4. Vaccine Hesitance is Anti-Science vs.Science Takes Time	INCLUDE: VETERANS' REFERENCES TO SCIENCE, RESEARCH, AND DATA AS WHAT THEY VALUE/RESPECT<br><br>Vaccine-hesitant patients see themselves as informed and advocates of true, unbiased science.<br><br>Veterans emphasize the need for large-scale, long-term research to understand the safety and effectiveness of vaccines.<br><br>With some exceptions, veterans felt that strong, longer-term data could change their minds.<br><br>Often, their complaints about the vaccine roll-out and vaccine promotion efforts were grounded in a sense that these were "not scientific" or didn't represent "good science."	Vaxxed:<br><br>1_26889;<br>1_33918;<br>1_34857;<br>1_41044;<br>1_14569;<br>1_17897;<br>1_28235;<br>1_37066;<br>1_39502;<br>1_42698;<br>1_43505;<br>1_46356;<br>1_47362<br><br>Unvaxxed:<br>1_30068;<br>1_30477;<br>1_38082;<br>1_38659;<br>1_25537;<br>1_33227	Vaxxed:<br><br><br>Unvaxxed:<br>2_9607;<br>2_18007;<br>2_31703;<br>2_37989;<br>2_38516;<br>2_37980	1_34857: "It just seemed to be fear-driven. Nobody knew anything about it…. Not only did nobody know anything really about COVID, the virus itself, but they knew even less about the vaccine. "; "That's not science. That's not medicine anymore. That's fear and that's control." [8:50]<br><br>1_41044: "I just think it's incredibly fast for there to have been human trials for a vaccine for something that just came out this quick" [4:35]; "I just wish that there had been a lot more time to research everything." [19:05]<br><br>1_14569: "That was my deciding factor why not to get it, just because I knew people who were still in the… test studies for it but then it was offered to the public<br><br>1_28235: "I trusted reading the science…. I looked at the statistics and things like that, and reading on the CDC website, reading on the different actual science websites that would tell me how the vaccine is supposed to be doing, and things like that." [2:05]<br><br>1_30068: [[not a quote]The vaccine was being pushed too hard by the government but hadn't been developed properly or safely. His involvement with research has answered a lot of his questions. He again states that it takes years to develop a vaccine and more years even to get approval. He believes they haven't had time to develop a functioning vaccine and you end up getting the virus when you get the vaccine. / Two to three years of research and development on the vaccine might make him more inclined to get the vaccine. It has to be proven, which it hasn't been yet. He has seen too much negative about the vaccine.<br><br>1_30477: "I definitely did not want to take a vaccine that had not been proven since I taught the scientific method and I could tell right away [inaudible] they were not proven" [00:12] / "The sources that, of course, I trust are the ones that the scientific	2_9607: "You know, I just don't, I don't, God didn't make me needing the flu shot, I might need summeric [drug name], I might need some olive oil in my diet or some grain bread or maybe, God didn't make me needing a Covid shot, he didn't make me needing a flu shot every year. That's my problem--you've got other problems with your immune system because you're not eating right, you're not exercising right, you're not doing what you need to do. I think medicine is bass-akwards. It's backwards-you're treating the symptoms instead of the problems. God didn't make me needing this stuff."<br><br>2_18007: "I don't know if this answers (your question) but, I'm data driven. So if I read an article (inaudible)…it's an opinion. If I read something like that, I take it with a grain of salt...for something like this, even if I don't understand it, I want the data."<br><br>2_31703: "Some of the European studies just didn't really follow a solid pattern with the science. It was very random, working and not working. The effects that were done, the early effects that were felt by the people who took it (the vaccine)"<br><br>2_37989: He feels the CDC thinks the everyday citizen is dumb. He doesn't think that rationale is true--he believes the average person is 'smarter about things.'<br><br>2_38516: "I watch the news and everybody says it's safe, it's safe, it's safe. But, like I said I was in the medical field and I do have a little bit of somewhat knowledge cause I worked in a hospital. Things don't always come out quickly and be the best. Sometimes they need to take a little time to get them perfected right"<br><br>2_37980: "There's a lot of data that's out there even if some of it is kind of couched, but, having, and I'll say this, having been in the medical profession myself there are things that I know and can see and can gleen that somebody else may not

Veteran	5. Vaccine Hesitance is Focused on Lack of Personal Risk and Apathy	INCLUDE: VETERANS' PERCEPTIONS THAT COVID VACCINATION MAY BE WORSE FOR PUBLIC HEALTH AND VULNERABLE POPULATIONS<br><br>There are reasons to be deeply skeptical of the public-health rationale for vaccination; by not getting vaccinated, I am protecting myself and others.<br><br>Veterans who chose not to get vaccinated did not necssarily do it because they felt that they themselves were healthy, strong, and at lower risk for COVID. While some felt this way, others described themselves as particularly vulnerable to severe disease.<br><br>Indeed, their concern was that, for them, and for other vulnerable populations, vaccination could be worse than COVID-19 disease.<br><br>While this stands in stark contrast with published research, many arrived at this conclusion based on their personal experiences and observations, and especially seeing vaccinated loved ones struggle with COVID after vaccination or struggle with vaccine side effects.	Vaxxed:<br>1_26889;<br>1_34857;<br>1_41044;<br>1_17897;<br>1_28235;<br>1_43505;<br>1_47362<br><br>Unvaxxed:<br>1_30068;<br>1_30477;<br>1_25537	Vaxxed:<br>2_33802;<br>2_30213;<br>2_44705<br><br>Unvaxxed:<br>2_9607;<br>2_37989;<br>2_2921;<br>2_21708;<br>2_38460;<br>2_46883	1_26889 - doubt vaccine effectiveness based on personal experience and watching family members who were vaccinated get more severe disease than those who were unvaccinated: [0:00:46] "I don't have any faith in the vaccine if you want to know the truth."<br><br>1_34857 - "Everybody that I know that got the shot got sick. Way sicker than I did." [3:15]<br>"I just felt a little under the weather for like 5 days…. Everybody else that I know that got the shot were sick as dogs for weeks and weeks and weeks. And I even know people that I worked with, their family members died - not that they died of the shot, but they died after they got the shot." [3:45]<br><br>1_41044: "My parents both got really really sick after they got the vaccine and the booster. And they don't get sick for any reason at all, they're very very healthy people." [3:20]<br><br>1_30068: "I do not believe the vaccine is safe. I believe that it's conspired by couple of different governments and basically to kill off older people [pause] because all my brother-in-laws, they all got the shot and they're dead now. My sister-in-law, she got the shot and she's dead and she got the covid after she got the shot and my father-in-law, same thing, he got the shot and died and he was healthy as a big dog. He walked miles every day, he swam miles and he got the vaccine and then three days later her got sick and two days later he was dead." [1:19]<br><br>1_30477: saw vaccinated famiyl members gets very sick and even some deaths in the family; sister became disabled after vaccination / lost her balance<br><br>1_43505: "I don't feel, with all the data that's come out, that the vaccine really helps	2_9607: She is not worried about being sick with Covid. She's already been hospitilized with Covid, survived and is not vaxed. Others she claims, have died from the vaccine or been very sick from it.<br><br>2_33802: "And that's why I didn't want to take the COVID shot because I figured it was a crisis that was created and then the bad man came up with the solution, which is the vaccine, so that's why I didn't want to take it."<br><br>2_37989: "I had read the ingredients and some of them are down right bad for your kidneys, even poisonious."<br><br>2_2921: "It hadn't proven to be truly effective. Plus, I didn't trust what was going on at the time. We were bombarded with what's real information and what's false information. You couldn't really put a finger on if this is really going to work or if we were being experimented on."<br><br>2_21708: "I had a heart condition and prostate cancer, so you can say my immune system is whacked out and I just don't trust the shot."<br><br>2_30213: "There's a lot of people with cardiac problems and then I start thinking about, well, and there's a lot of people that have [inaudible] young and healthy and then having cardiac problems. But, then they don't even question whether or not it was related to vaccines or COVID. I think that's interesting."<br><br>2_38460: "One of my friends that did not get the vax got covid and they put her on ivermectin and three days later they put her on Ivermectin and she was really well...One relative and one friend who had the vaccination both died."
Provider	1. Info Readily Available vs. Basic Questions Unanswered	To get veterans on board with vaccination, give them the facts.<br><br>Relevant information on vaccine risks and benefits easy to access. The science is clear, and high-quality information about vaccines is available.<br><br>If and when veterans are approaching the topic rationally, it is a question of just conveying the available information more clearly and consistently and dispelling misinformation.<br><br>What was unsaid here was more important as what was said: no one shared veterans' perspective that there are significant gaps in available data; though many did feel that information could and should be better conveyed.<br><br>In short, it's not that new information or data are needed; it's just that we need to better share that info and educate people regarding existing data.	Intervention:<br>1_007;<br>1_009;<br>1_011;<br>1_001;<br>1_003<br><br>Control:<br>1_010;<br>1_012;<br>1_014;<br>1_015;<br>1_016;<br>1_018	Intervention:<br>2_001;<br>2_002;<br>2_004;<br>2_007;<br>2_013<br><br>Control:<br>2_018;<br>2_009	1_007: "We have weekly director's messages to veterans that include information on that [vaccination], we have done a multitude of outreach events… including going into the community… all of our PACT Act registration outreach events also offered vaccinations as a part of that" [11:18]<br><br>1_010: "Sometimes you have to try everything for certain people. You have to explain the dosage. You have to explain the type of strand that it can cover now. You have to promote the fact that it's better than it was before, it covers a wider range, those kind of things. Then you kind of have to use family and friends - you want to protect them." (12:31)<br><br>1_011: "When we do nursing appointments we talk about vaccinations as well and that gives me the time, I always take however long the Veteran needs to answer all their questions and concerns." [20:20]<br><br>1_011: "I think that just education is the number one thing. I mean if you can show the advantages to people if it is getting COVID or any other vaccine … I think the success rate will always be high." [33:05]<br><br>1_012: "I think explaining them in a positive way and giving the data that how many vaccines have been provided and how it has helped people [inaudible] from covid infection, if we show the proper data, if we have this data in front of them, instead of just verbally telling, if it's printed and given to the vets with examples, I think that would help more because if its visualized and they can see the numbers that will help them more to understand" [9:40]<br><br>1_014: "The most effective method that I would think of is just more education" / "Just have the information readily available, have it packaged in a way that's easily	2_001: "we created a program called 'mythbusters' which was basically just - literally googled what the negative things were online being said about this, that, and the other and all the vaccines and we did 'mythbusters'." (2:34)<br><br>2_001: "Education, education, education, we need to own it."<br><br>2_002: "It's, you know, just important to provide them with the facts, you know, the research, the evidence based science that's come out and that kind of thing." (2:38)<br><br>2_002: "Information, you know, just informing some of the veterans that it is (the COVID vaccine) medically recommended in most situations"<br><br>2_004: "I think it's still just you know, I think its just a lack of education. Providing more education about the actual virus and then the vaccine and helping people understand it better"<br><br>2_009: "The vaccine has been around long enough now that people really don't - I dont feel like they're questioning the safety anymore, it's just remaining a political issue." (6:51)<br><br>2_013: "I think that if you could show the facts and really have really solid facts, I believe that that would help with people in general just being reluctant, especially veterans, being reluctant to taking the vaccination." (15:29)

Provider	2. Conspiracy Theories vs. Legitimiate Grounds for Doubt and Mistrust	Misinformation, propaganda, and conspiracy theories drive mistrust and vaccine hesitance.<br><br>If there are safety concerns, they are often not reality-based or fact-based.<br><br>Vacine concerns are described with a proverbial roll of the eyes and generally dismissed as "tin-foil hat stuff," "fake narratives," and "silly conspiracies."	Intervention:<br>1_004;<br>1_007;<br>1_008;<br>1_009;<br>1_011;<br>1_017;<br>1_001;<br>1_003<br><br>Control:<br>1_014;<br>1_015;<br>1_016;<br>1_019	Intervention:<br>2_001<br><br>Control:<br>2_018;<br>2_019;<br>2_006<br>2_012	1_004: "I have had multiple conversations with veterans and I think that false information is really detrimental and has influenced veterans in choosing not to get that vaccine."<br><br>1_007: "I think the top issue is probably something everyone faces with misinformation and disinformation that is publicly and widely available."<br><br>1_008: "What I've noticed is we kinda have quite a hesitant population and there seems to be a lot of misinformation. So staying current and abreast of reports. There's a lot of things like 'oh, it gives you blood clots, it does this, it does that' … So staying abreast of alot of the misinformation about complications about the vaccine itself." [2:15]<br><br>1_008: "Once patients became misinformed about one vaccine, they weren't going to get any vaccines at all. And really it's about finding the right opening and having the conversation about how vaccines work, and then why they're valuable, and sort of dispelling a lot of the misinformation that's out there. So that's the approach I've taken and I've tried to help my adult practioners take as well." [2:43]<br><br>1_009: "Literally, if the VA got rid of social media, I think that you'd be better." [13:00] … "I mean anyone can say anything on social media or twitter or tiktok and it's golden and it's believable. You don't have to do any research anymore, right. I just have to go on Twitter and listen to what someone else is saying and that's golden today." [13:07]<br><br>1_011: " One of the reasons I heard was because it had rattlesnake venom in it. Another reason I heard is because it had a tracking device in the immunization so that the government was tracking them." [5:54]	2_018: "they are scared of some effect on DNA, they got some message from somewhere."<br><br>2_018: "Maybe they need a clear-cut literature or something that this vaccine is not affecting anything…. So I think something clear-cut information, something literature to prove, that's maybe helpful, that we can give to them." [5:52]<br><br>2_018: "we have to give more education, educate them." [12:14] ... "I think maybe more literature ... proving that there is no risk for anything. Especially for the youngsters." [12:40]<br><br>2_018: "I think there is no training problem. I think they know but to get this information to the patients is the hard problem." [19:47]<br><br>2_019: "I don't see where they could do more. I mean they have it running on the little videotrons in the lobby. They have like you know about COVID and the rate of it how it's growing ... getting vaccinated vs not getting vaccinated." [19:09] "I mean I just don't see where more could have been done. " [19:26]<br><br>2_006: "There's mistrust, and, you know, there's silly consipracies that there's a chip put in it, or - I've heard all kinds of things." (12:37)<br><br>2_012: "Well, there's a lot of misconceptions about the vaccine and the side effects." (8:39)<br><br>2_012: "Covid just had so many questions about where it originated from and what it was - it's very hard to overcome those fears. And, the misinformation that is out there about COVID, it's very hard to - it's very hard to get a patient to understand
Provider	3. Vaccine Safety Concerns Irrational vs. Rational	Patients' vaccine concerns are primarily rooted in politics and emotions.<br><br>Concern about side effects is even described as paranoia.<br><br>They are stubborn and set in their ways; their concerns are not rational.<br><br><br>Related:<br>They generally distrust the government and authorities. (Very few people brought up that there might be some grounds for this mistrust, in big contrast to the vets.)	Intervention:<br>1_008;<br>1_011;<br>1_017;<br>1_003;<br>1_005<br><br>Control:<br>1_014;<br>1_019	Intervention:<br>2_001;<br>2_003;<br>2_005;<br>2_007;<br>2_008;<br>2_012;<br>2_013<br><br>Control:<br>2_006;<br>2_009;<br>2_020;<br>2_022	1_008: "this has become so politicized that some people just take it as a, I mean I hate to say it, but as a big government conspiracy, and it becomes an emotional issue rather than something that's rational uh rationally evaluated."<br><br>1_011: "I think it's a trust factor and many times a lot of these are mental health folks that are out and homeless as well. So some of them already have the paranoia, I mean that's just part of the mental health diagnosis that they have. And I think that's part of it and it's a big trust factor." [10:22]<br><br>1_014: "I guess to some extent the biases that have built up you know whether you can attribute [corrected from "contribute"] some to political or other factors" [6:28]<br><br>1_017: "That's the thing, a lot of them are not educated [at] our site. Some of them doesn't really know the difference [between the Flu and COVID-19]." (22:48)<br><br>1_019: "We're at that point with the people who just are not going to get it come hell or high water." (7:10)<br><br>1_019: "It's been so politicized from the start, at least where we are." (9:26)<br><br>1_019: "I don't know what it would take to change their mind, honestly. Because they are so committed to that side of it, their side." (9:58)<br><br>1_003: "The people who are rabidly anti-vax, it's going to be hard to get them no matter how much motivational interviewing you do."<br><br>1_005: "There's been very few patients in recent months that are interested in learning more about COVID vaccines. So a very large amount have kind of made up their minds	2_001: "We're a rural state, our CBOCs of course are in rural areas. I think a lot of it in the beginning was political. We are a republican state, and I do think that really affected us. Their misinformation I was hearing I was being quoted stuff from Breitbart and Fox everyday [inaudible] really hard to combat because you don't want to get in those kinds of conversations with a veteran." (8:31)"<br><br>2_003: "It's mentality. Sometimes, people are set in their ways and they have beliefs, and these beliefs are unbreakable." (10:07)<br><br>2_003: "These are beliefs that they have construed and they believe them, so that is a barrier because it's very hard once something is set in to a persons mind and they believe in this whole-heartedly, to break that barrier. And, it takes alot of education." (10:39)<br><br>2_003: "I think just the initial fear. Anytime it's something new, fear incites people, and just the initial fear. It's very hard to get around." (12:28)<br><br>2_005: "I have some [patients] that are very, very stuck in their ways. And, I don't know that we'll ever talk them into it, unfortunately. I really have no answer for that one." (7:00)<br><br>2_006: "They don't trust it… There's just a mistrust, a general mistrust with the whole thing." (13:47)<br><br>2_007: "I think the challenge would be, I guess their mindset, it's hard to - once they have their minds set on something it's hard to change their mind on it." (6:00)<br><br>2_009: "The big issue that we face here was that it was so politicized that those that

Provider	4. Vaccine Hesitance is Anti-Science vs. Open to Science	Vaccine hesitant patients don't trust or understand science.<br><br>They believe everything they read on the internet and are not critical consumers of information.<br><br>Confusion and ignorance are widespread.<br><br>You can't really get through to this group with rational explanations.	Intervention:<br>1_008;<br>1_011;<br>1_017;<br>1_001<br><br>Control:<br>1_012;<br>1_015;<br>1_019	Intervention:<br>2_001;<br>2_003;<br>2_005;<br>2_008;<br>2_012;<br>2_013<br><br>Control:<br>2_006;<br>2_009	1_012: "Some of the vets who are like pre-determined that they do not want or they do not want to listen about any vaccine... sometimes its very difficult when you are pre-determined or you already made your choice or decision that you do not want to get that vaccine, it's difficult to explain them or make them understand."<br><br>1_015: "In the CBOC that I work at, because it's very rural, we have a large population who still believe it's not true. They just don't believe in the COVID pandemic and they dont believe that the vaccine is necessary or it's safe." (5:00)<br><br>1_015: "... how to deal with those patients who are very vocal in their beliefs of all the conspiracy theories that they hear on the inter - that they read about on the internet." (7:57)<br><br>1_017: "Even though how much we educate them. It's still hard to convince them." (12:01)<br><br>1_019: "If I'm honest, we need to cut off all of their news channels and their access to the internet." (9:15)<br><br>1_019: "I don't know what it would take to change their mind, honestly. Because they are so committed to that side of it, their side." (9:58)<br><br>1_001: "With the advent of social media, a lot of times, what they will see on some of the social media networks and channels and things like that, they'll come back saying 'well, I saw on the news that it's not really effective,' or 'I saw that this person had this bad outcome, so I'm definitely not going to get the shot.' So those are kind of the things, unfortunately, we have difficulty controlling because that's all out there in social media. So trying to change that by getting the word out on the other hand,	2_003: "I put a lot of emphasis on the African American race because a lot more education is probably needed in those areas. Simply because of the lack of knowledge." (24:58)<br><br>2_003: "if you're not informed and you honestly don't understand the process - the disease process, then you're not going to get [it]"<br><br>2_005: "Just people kind of stuck in their ways and unwillingness to listen to science versus whatever they've heard from Neighbor Joe down the street." (6:15)<br><br>2_008: "Pretty much, some have made up their mind that it [COVID-19] doesn't exist or it's not something that they want to believe in."<br><br>2_008: "Some of them will look it up on the internet or Facebook, those kind of places to get the whole wrong idea about what the vaccine is supposed to be for - to begin with." (7:11)<br><br>2_012: "If we have a patient who has not had the primary series, they are pretty much not going to be convinced to take it." (9:35)
Provider	5. Vaccine Hesitance is Focused on Lack of Personal Risk and Apathy	If you want to appeal to vets, emphasize their own vulnerability to severe disease.<br><br>They may think they are healthy and strong so they don't need to be vaccinated.<br><br>In some cases, they may just be just tired of hearing about COVID and don't think it is really relevant to them.<br><br>Something has to "scare them" into choosing to get vaccinated.	Intervention:<br>1_006;<br>1_017<br><br>Control:<br>1_010;<br>1_013;<br>1_016;<br>1_018;<br>1_019	Intervention:<br>2_010;<br>2_016;<br>2_021<br><br>Control:<br>2_006;<br>2_014;<br>2_022	1_006: "The COVID fatigue of hearing about it, seeing about it. A lot of people are like, 'I don't care anymore. I've been here 3 years, move on.' So I think those conversations have been difficult." [6:55]<br><br>1_010: "Now it is a challenge because their reasoning is 'the world has it, it's opened up, we don't need the vaccine, I had COVID I am not going to get it again'. You know, various comments, it is challenging." (9:20)<br><br>1_013: "If we encourage--hey, you need the shingles vaccine, 'ok, I can get it'... but for the COVID, they don't care."<br><br>1_013: "They think they don't need the vaccine anymore." / "We have to live with COVID."<br><br>1_016: "I hear a lot of patients just - they just don't seem to think COVID's a very big deal anymore. So, a lot of it unfortunately is tied to misinformation." (7:25)<br><br>1_017: "We go for a fear factor kind of thing. We base it on mortality, we base it on statistics.... We give them examples." (12:59)<br><br>1_018: Veterans "are no longer coming in and wanting to talk about it because there's nothing reminding them that this [COVID] is still a thing."	2_006: "They think that COVID's over, so, they're not interested in vaccinations." (7:28)<br><br>2_010: "I think they think it's over with the masking thing not required any longer. I just don't think there's that kind of urgency any longer." (5:10)<br><br>2_010: "I don't want to say a scare tactic, but you know, this is what can happen if you don't get it." (7:47)<br><br>2_014: "Sometimes when you - patients they look around them and they feel like we're not masking up anymore, the pandemic is kind of winding down, so they feel like there's not neccessarily a need to be vaccinated if they haven't already done so." (9:05)<br><br>2_014: "When you kind of put it in their minds that the COVID is still out here and that they do have health issues or chronic health concerns that will put them at a higher risk of having a severe infection if they are infected. It kind of maybe changes their perpective a little bit because they know that they are at a higher risk." (9:20)<br><br>2_016: "the main thing is the weariness, that they are just over it. They do not want to deal with the vaccines any more, since we have had so many in such a brief short period of time. I think that is truly the main thing, that they are just tired of it. Which I get it."<br><br>2_021: "You know their loved one or best friend or whatever gets really sick or passed away I think more people would be inclined to. But, unless it's something like that, I just don't know how we can honestly motivate them anymore. You know a lot of them has just got their mind made up; yes I will or no I won't." [21:50]
